# Impacts of Anthropometric, Biochemical, Socio-Demographic, and Dietary Habits Factors on the Health Status of Urban Corporate People in a Developing Country

**DOI:** 10.3390/healthcare8030188

**Published:** 2020-06-27

**Authors:** Masuda Begum Sampa, Md. Rakibul Hoque, Md. Nazmul Hossain

**Affiliations:** 1 Advanced Information Technology, Kyushu University, Fukuoka 819-0395, Japan; 2 School of Business, Emporia State University, Emporia, KS 66801, USA; mhoque@emporia.edu; 3 Faculty of Business Studies, University of Dhaka, Dhaka-1000, Bangladesh; nhossain01@du.ac.bd

**Keywords:** urban corporate people, blood uric acid, developing country, health status, multinomial logistic regression

## Abstract

This study focused on urban corporate people and applied multinomial logistic regression (MLR) to identify the impact of anthropometric, biochemical, socio-demographic and dietary habit factors on health status. Health status is categorized into four levels: healthy, caution, affected, and emergent. A cross-sectional study, based on convenience sampling method, was conducted to select 271 employees from 18 institutions under the Grameen Bank Complex, Dhaka, Bangladesh. Biochemical measurements such as blood uric acid are highly significant variables in the MLR model. When holding other factors as constants, with a one-unit increase in blood uric acid, a person is 11.02 times more likely to be “emergent” compared to “caution”. The odds are also higher, at 1.82, for the blood uric acid to be “affected” compared “caution”. The results of this study can help to prevent a large proportion of non-communicable diseases (NCDs) by reducing the most significant risk factor: blood uric acid. This study can contribute to the establishment of combined actions to improve disease management.

## 1. Introduction

Anthropometric and biochemical measurements are important factors for determining the health status of an individual. These are also used to diagnose chronic illness [1]. Some previous studies confirmed the impact of socio-demographic characteristics and dietary habit on the health status [2]. The extent to which anthropometric, biochemical, socio-demographic, and dietary habit factors have an impact on health status has been investigated, but the results are still debatable. Moreover, relatively little research has focused on the combined effect of anthropometric measurements, biochemical measurements, dietary information and socio-demographic characteristics on health status in developing countries like Bangladesh. Consequently, it is important to examine these factors simultaneously to assess their importance, particularly in urban corporate people. The urban population in developing countries is increasing because of rural urban migration. Currently, about 39% people are living in urban areas of Bangladesh; this was only 10% in 1975. The majority of the working-age group living in the urban area of Bangladesh have significant workloads and remain seated for a long time to complete their tasks. Besides, little chance exists to engage in physical activities among the urban people in Bangladesh because of a lack of playgrounds, parks, walkable footpaths, and safe roads for cycling. Due to lifestyle differences, the prevalence of non-communicable diseases (NCDs) is also higher among urban than rural people in Bangladesh [3]. Each year, NCDs kill 41 million people, equivalent to 71% of all deaths worldwide. About 15 million deaths due to NCDs occurred in the working-age groups. Over 85% of these "premature" deaths occur in low- and middle-income countries. NCDs threaten the progress of the 2030 Agenda for Sustainable Development Goals (SDGs), which includes a target of reducing one third of premature deaths from NCDs by 2030 [4].

Like other developing countries, Bangladesh is changing in the disease and death patterns from communicable diseases to NCDs and due to urbanization and economic growth, these are spreading very fast here [5,6]. Moreover, communicable diseases are becoming life threatening due to NCDs. Diseases that are communicable and non-communicable have been recognized as a threat for young people too [7]. The prevention and control of NCDs depends on the availability of information about the risk factors. In most of the developing countries, NCDs are detected late, and patients need extensive and expensive medical care for severe complications [8]. 

People who usually work in urban areas of Bangladesh lack health insurance and high health awareness, do not get routine mandatory health checkups and are not habituated to using ICT-based healthcare services [9]. It is also necessary to examine the specific role of each risk factor on the health status of a specific group of people or a given setting to consider in-depth planning, implementation, and evaluation of health programs by using good policies to prevent and control diseases [10,11,12,13]. Thus, we aim to identify the predictors of health status and to estimate the relative contribution of each anthropometric variable, biochemical measurements, dietary information, and socio-demographic characteristics of health status. This study addresses the following research questions:

- How much does each factor contribute to the health status among urban corporate people in Bangladesh?
- What is the most significant factor for influencing the health status among urban corporate people in Bangladesh?

## 2. Materials and Methods 

### 2.1. Hypotheses Development

The health status of an individual can be measured by previous and current diagnosed illnesses and by clinical parameters [14]. Diseases like communicable and non-communicable diseases (NCDs) have become the leading cause of deteriorating the health status resulting in morbidity, disability and mortality globally [8]. The causes for developing NCDs and mortality due to NCDs vary across different places and regions as well as different populations [8,15,16]. In the following section, we will describe the socio-demographic characteristics, dietary habits, anthropometric measurements, and biochemical measurements as they may relate to the health status of an individual. 

#### 2.1.1. Socio-Demographic Characteristics

Socio-economic status plays an important role in the determination of health status [17]. Studies have confirmed the profound impact of socio-demographic characteristics (age, gender, education level) on health status [2]. 

Therefore, we hypothesize that the higher the level of education, the better the health status. We also expect that the health status will worsen with age. Health status may be good or bad for males or females, but we have no prior expectations. 

#### 2.1.2. Dietary Habits

Dietary habit is associated with different clinical measurements and has an influence on health status [18]. Due to rapid urbanization and lifestyle changes, people are more likely to eat typical fast foods outside such as pizzas, hamburgers, deep-fried foods (e.g., Singara, Samosa, Mughlai Paratha, etc.). and sugar-containing drinks in Bangladesh. These outside foods in Bangladesh are not hygienic. However, they are gaining popularity and many fast food shops are operating in urban areas [19].

Therefore, we hypothesize that dietary habits (drinking sugar-containing drinks and eating fast foods) have a bad impact on health status.

#### 2.1.3. Anthropometric Measurements

Anthropometric measurements such as weight, height, body mass index (BMI), body circumference (arm, waist, hip, and calf), waist to hip ratio (WHR), elbow amplitude and knee–heel length, etc., are highly related to the health status of an individual [20]. They reflect the health status of an individual and also predict the overall performance of the health and survival of an individual [21]. Additionally, these measurements are used as a baseline for physical fitness and to measure the progress of fitness [22]. 

Therefore, we want to explore how much of an impact anthropometric measurements have on health status.

#### 2.1.4. Biochemical Measurements

Biochemical measurements such as blood uric acid are used to assess the health status of an individual. Poorer health status is associated with higher blood uric acid. High uric acid is a biomarker of various chronic diseases and thus reflects one’s health status [23].

Therefore, we want to explore how much of an impact this biochemical measurement has on health status among urban corporate people.

**Figure 1:**
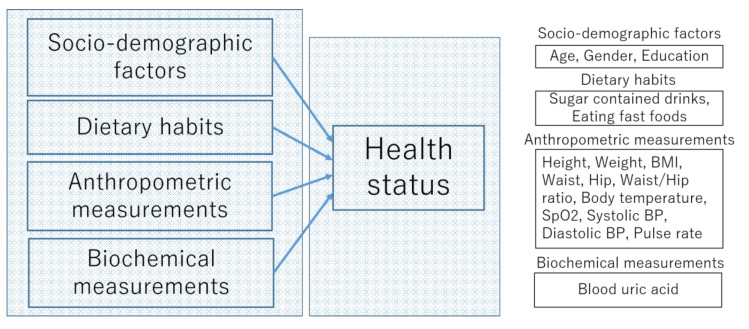
The conceptual framework of the multinomial logistic regression (MLR) model.

The conceptual framework of the multinomial logistic regression (MLR) model is shown in Figure 1.

### 2.2. Study Place

A cross-sectional study was conducted among all employees who agreed to participate in the portable health clinic (PHC) health checkups (described in the next section) and eHealth services in the Grameen Bank Complex in Dhaka, Bangladesh. The Grameen Bank Complex holds 18 different offices, such as Grameen Bank, Grameen Communications, other non-government organizations, and private companies, with more than 500 workers.

### 2.3. Portable Health Clinic (PHC) System

**Figure 2:**
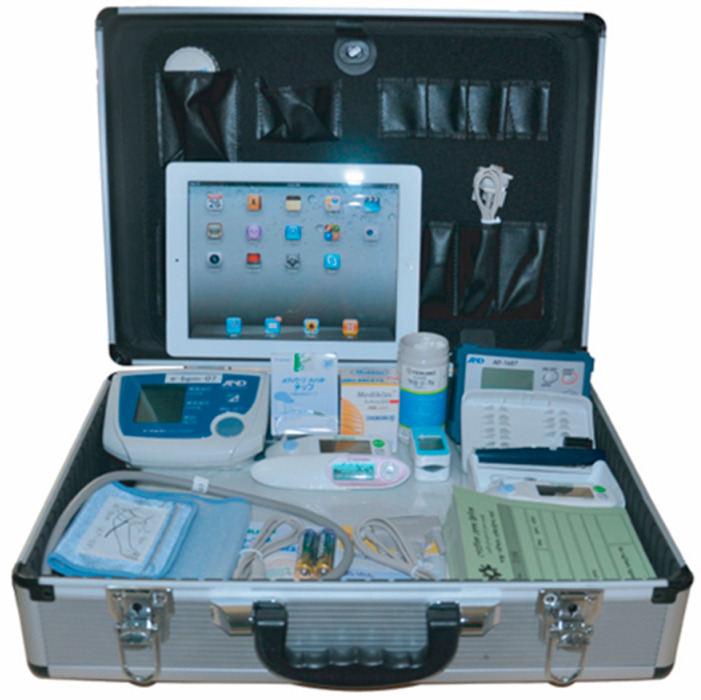
Portable health clinic (PHC) box with different medical sensors.

Grameen Communications, Bangladesh, and Kyushu University, Japan, have jointly developed a human-assisted PHC system [24]. A PHC is an eHealth system that aims to provide affordable primary healthcare services to prevent severity or to control non-communicable diseases (NCDs). A PHC system has four modules: (a) a set of medical devices (shown in Figure 2), (b) a software system to collect and archive medical records, (c) healthcare workers to make the clinical measurements and explain ePrescriptions, and (d) ICT-trained call center doctors. Consumers come to the service point, and a health checkup is conducted by pre-trained healthcare workers. If needed, the consumer is connected to the call center doctors for a consultation. The clinical measurements addressed by a PHC are as follows: (1) blood pressure, (2) pulse rate, (3) body temperature, (4) oxygenation of blood (SpO2), (5) arrhythmia, (6) body mass index (BMI), (7) waist, hip, and waist to hip ratio, (8) blood glucose, (9) blood cholesterol, (10) blood hemoglobin, (11) blood uric acid, (12) blood grouping, (13) urinary sugar, and (14) urinary protein. 

In this study, the test items included (except arrhythmia, blood cholesterol, blood hemoglobin, blood grouping, urinary sugar, and urinary protein, asthere were many missing and outlier cases in these measurements because multinomial logistic regression does not consider outlying cases [25]) in this PHC system were used as predictors of health status, which is set as an outcome variable. 

### 2.4. Data Collection

Before the implementation of PHC services, awareness events and prior notification to each employee were made to provide information on the availability of PHC services. Data was collected from 6 August 2018 to 16 August 2018 through a convenience sampling method from a sample of 271 employees who came to the PHC service center inside the Grameen Bank Complex to receive the PHC service. All participants involved were treated based on the safety procedure described in the “Helsinki Declaration 2013” [26]. The data were secured at the data center, and participants’ privacy is protected. Only researchers, data entry officers, and data managers could access the data with a secure password. 

Socio-demographic and dietary information were collected using a questionnaire. Each individual’s clinical measurements such as height, weight, BMI, waist to hip ratio, body temperature, blood glucose, pulse rate, and blood uric acid were measured by pre-trained healthcare workers. The descriptions of independent variables are presented in Table 1.

### 2.5. Health Status

Health status is an important outcome or method for assessing the level of health of a person, a group, or a population, as calculated by the individual or by objective measures [27]. Health status is assessed from one’s anthropometric measurements including blood pressure, BMI, pulse rate, and blood uric acid, etc. [23]. In this study, health status is assessed depending on the results of health checkup items provided by the PHC service [28]. We used the “Bangladesh-logic (B-logic)” risk stratification (which was created based on the international diagnostic standards) technique to rank the risk levels of each health checkup item result into four categories: healthy, caution, affected, and emergent [2,22,29]. The overall health status of each subject was determined by the worst level of all health checkup items [2]. Therefore, the health status is divided into multiple categories based on the results of health checkup items. The four levels of health status are, 1=healthy, 2=caution, 3=affected, and 4=emergent. Examples of the determination of health status or overall health conditions based on the results of each health checkup item are shown in Table 2.

### 2.6. Data Analysis Technique

Multinomial logistic regression (MLR) analysis was applied (by using R statistical software) to analyze the association of clinical factors, dietary information and socio-demographic factors with the health status. MLR is an extension of the binary (or dichotomous) logistic regression and it is used when the outcome variable has more than two outcomes. For the MLR, one category or level of the dependent variable is specified as the reference category and regression coefficients are estimated for each independent variable [30]. The independent variables of MLR can be of a nominal, ordinal, or continuous type [31]. Multinomial logistic regression uses a maximum likelihood estimation method. It also uses multiple equations [32].

### 2.7. Ethical Approval

The authors obtained ethical approval from the National Research Ethics Committee (NREC) of the Bangladesh Medical Research Council with approval No. 18325022019. 

## 3. Results

### 3.1. Descriptive Statistics

Descriptive statistics were used to describe the baseline characteristics of the participants. The summary statistics of participants are shown in Table 3 and Table 4. 

The mean age of participants is 49.61, most of the participants are aged 50 years. About 83% of respondents are male and most of them have completed a college/university degree. Among 271 respondents, 9.6% reported that they drink sugar-containing drinks (Coke, Fanta, soda, fruit juice) three or more times a week and 18.1% reported that they eat fast foods (pizzas, hamburgers and deep-fried foods) three or more time a week. Among 271 employees 2 (0.7%) reported as “healthy”, 80 (29%) reported as “caution required”, 122 (45%) reported as “affected” and 67 (25%) reported as “emergent”.

According to BMI measurements, the mean value of BMI = 25.37, i.e., most of the participants are overweight. According to the WHO, a BMI ranging from 25 to 29.9 is defined as overweight. The mean amount of uric acid is 6.63 mg/dL. This means that the uric acid of most of the people is close to the borderline of the suggested normal range: <7.0 mg/dL [33]. 

### 3.2. Multinomial Logistic Regression Estimation

Multinomial logistic regression (MLR) analysis by using R statistical software was applied to estimate the influence of health checkup test results, including blood pressure, pulse rate, and blood uric acid, on health status. MLR does not assume a linear relationship between the dependent and independent variables [34], and there are studies that have generated dependent variables from independent variables [35]. For the “healthy” category, the number of cases is only two (0.7%) which is not representative of the total sample size of 271. The sufficient size of the smallest multinomial category is a factor of the predictive performance of the MLR model. MLR estimated by maximum likelihood yields over-fitted prediction models in small- to medium-sized data [36]. If a category has very few cases, the model may become unstable [37]. This is why we omitted the “healthy” category from the further MLR model. Therefore, two model equations are estimated from three categories: “affected” relative to "caution”, and “emergent” relative to “caution” whereas the reference category is “caution”. The first equation comparing health status = "affected" to the baseline health status = "caution" and the second equation comparing health status = "emergent" to the baseline health status = "caution". If we consider coefficients from the “affected” row to be b1 and coefficients from the “emergent” row to be b2, we can write two model equations:

ln(P(health status = affected) /P(health status = caution)) = b10 + b11X1 +…+ b1iXi(1)

ln(P(health status = emergent) /P(health status = caution)) = b20 + b21X1 +…+ b2iXi(2)

where b1i is a regression coefficient associated with the ith explanatory variable and the first level (affected) of the outcome variable and b2i is a regression coefficient associated with the ith explanatory variable and the second level (emergent) of the outcome variable.

#### 3.2.1. Relative Risk Ratio

The ratio of the probability of choosing one outcome category over the probability of choosing the baseline category is referred to as the relative risk ratio. The relative risk ratio can be obtained by exponentiations of the logit coefficients. 

#### 3.2.2. Multicollinearity Check

The standard errors of the coefficient estimates (B) are used to check for numerical errors or multicollinearity in the solution of the MLR. A standard error that is greater than 2.0 indicates that multicollinearity exists among the independent variables [30]. 

#### 3.2.3. Parameter Estimation

Table 5 summarizes the results of the multinomial logistic regression analysis for the association between independent variables and the health status (dependent variable). The results for fitting the MLR models are given in Table 5, which also shows the coefficient estimates (B), the standard errors and the odds ratio (OR), represented by Exp(B), for each level of health status. First, the coefficients for “affected” in comparison to the baseline category, which is “caution”, and then the coefficients for “emergent” in comparison to the baseline category, which is “caution”, is represented in the Table 5.

#### 3.2.4. Interpretation of the MLR Results

The output coefficients are represented in the log of odds. Relative risk ratios allow for an easier interpretation of the logit coefficients. They are the exponentiated values of the logit coefficients. 

Gender is a significant non-policy variable that increased the likelihood of one being “emergent”. The estimated coefficient is negative, which indicates that females are more likely to be “emergent” than males. Blood uric acid is a highly significant variable in the MLR model. The estimated coefficient is positive, which indicates that employees with high uric acid are more likely to become “affected” and “emergent”. Education, drinking sugar-containing drinks, eating fast foods, body temperature, and pulse rate fail to be significant explanatory variables; the results do not provide evidence about a link between health status and these variables.

Looking at the blood uric acid, it can be concluded that, when holding other factors constant, for a one-unit increase in blood uric acid, the person is 11.02 times more likely to be “emergent” compared to being “caution”. The odds are also higher, at 1.82, for the blood uric acid to be “affected” compared to being “caution”. Respondents are significantly more unlikely to being “emergent” in terms of height, waist and BMI. This is because the odds ratios of the category “emergent”, when compared to “caution”, are close to zero for these factors. Therefore, there is a huge difference between the category “emergent” and the “caution” for these three factors. The estimated coefficient of BMI is negative (−2.77), which indicates weight is higher compared to height. Those who have a higher weight compared to height tend to be “emergent” compared to “caution”. The negative estimated coefficient of height (−0.91) means shorter people tend to be “emergent” compared to “caution”. On the other hand, respondents are significantly more likely to be “emergent” compared to “caution” on weight, hip, WHR, and systolic blood pressure (systolic BP). The positive estimated coefficient for hip (0.42) means those who have a high hip circumference tend to be “emergent”. The positive estimated coefficient of WHR means those who have a high waist to hip ratio tend to be “emergent”. The positive estimated coefficient of weight means people who have more weight tend to be “emergent”. Respondents are significantly more likely to be “affected” compared to “caution” in terms of their waist, and diastolic BP.

## 4. Discussion

This is the first study in the context of a developing country like Bangladesh that reports the predicting factors of levels of health status among urban corporate people. This study has found that, in an urban corporate setting, females are more likely to be vulnerable than males. Therefore, this finding will help us to warn female corporate workers to be more concerned about their health status. By our analysis of the multinomial logistic regression model, we have shown that blood uric acid appeared to be the best predictor of health status compared with other parameters, while BMI was the weakest predictor. A purine-enriched diet, obesity, and alcohol intake are reported as the predictors of hyperuricemia (high blood uric acid) [38,39,40]. Approximately two-thirds of the uric acid is derived from the metabolism of endogenous purine, and the remaining as a result of eating purine-enriched foods [41,42,43]. Due to economic improvement and lifestyle changes, the tendency of eating purine-enriched foods such as lentils and red meat is high among urban people in Bangladesh. The chance to engage in physical activities is also low among urban people in Bangladesh. There are very few studies that have reported the impact of NCD risk factors on health status. However, previous studies identified the prevalence of different risk factors of NCDs in different places. For example, being overweight was more common in urban than rural areas in a previous study [12]. Waist–hip ratio appeared to be a stronger risk factor for NCDs such as myocardial infarction, stroke, and premature death [44]. 

Countries like Bangladesh need to make proper policies for each risk factor separately to prevent the harmful prospects of them [6]. Priority actions for the most impactful risk factors in the limited budget will bring enormous benefits to social and economic development and the health sector, and it will be helpful in lowering the prevalence of the major risk factors among the population [45].

This study worked with a human-assisted portable health clinic system, with the aim being to provide primary healthcare services, including blood uric acid measurement, regularly. Regional and corporate officials who have a high risk of developing NCDs must be investigated in terms of the effects of socio-demographic characteristics, dietary habits, anthropometric, and biochemical measurements on health status. One of the strengths of our study is the identification of the most significant risk factor of corporate people in Bangladesh who have a high risk of developing NCDs and do not have national health insurance. Another strength is that all clinical measurements were done using cost-effective PHC instruments by pre-trained healthcare professionals rather than being self- reported by the participant. The results of this study can help to prevent a large proportion of NCDs by reducing the most significant risk factor: blood uric acid. This study can contribute to the establishment of combined actions to improve NCD management.

## 5. Limitations of the Study

This study is limited by geographical scaling; the survey has been conducted in one country and focused on 18 institutions only. Thus, the results may raise a concern about the generalizability of the findings. Therefore, there is a scope to make its findings more generalized by examining more institutions and countries. A future study could include additional factors such as physical activity, information about eating purine-enriched foods such as red meat, lentils and sodium-containing foods, mental health factors, and environmental factors (duration of living in a city)**.**

## 6. Conclusions

This work assessed the importance of risk factors to predict the health status of urban corporate people in Bangladesh. Anthropometric variables, socio-demographic characteristics, dietary habits, and biochemical measurements were used in the multinomial logistic regression model. Data were collected from 271 employees (46 female) aged between 34 and 77 years. Among urban corporate people, blood uric acid was found to be the factor that was most significantly (high impact variables) associated with health status. Interventions to reduce these factors are needed and should target older employees, who have a high waist to hip ratio (i.e., the waist circumference divided by the hip circumference) and a large hip size, large waist size and high levels of blood uric acid. 

Screening and health promotion initiatives should be launched by targeting the high-impact risk factors for health status. However, to prevent the seriousness of NCDs, all of these risk factors should be measured and monitored regularly. This study can contribute to the establishment of combined actions to improve NCD management. Creating employees’ clubs or employees’ associations can help to improve the health status, wellbeing of employees and engage them in physical activities.

## Figures and Tables

**Table 1:** Description of independent variables.

Number	Independent Variables	Description	*n*
	**Clinical factors**		
1	Height (cm)	Height of the participant	271
2	Weight (kg)	Weight of the participant	271
3	BMI (kg/m^2^)	Weight divided by the square of the height	271
4	Waist (cm)	Waist circumference	271
5	Hip (cm)	Hip circumference	271
6	Waist/hip ratio	Waist to hip ratio	271
7	Body temperature (°F)	Body temperature	271
8	SpO2	Oxygenation of blood (%)	271
9	Systolic BP (mmHg)	Systolic blood pressure	271
10	Diastolic BP (mmHg)	Diastolic blood pressure	271
11	Pulse rate (bpm)	Pulse rate	271
12	Blood uric acid (mg/dL)	Blood uric acid	271
	**Socio-demographic factors**		
1	Gender	Gender of the participant	271
2	Age	Age of the participant	271
3	Education	Education completed by the participant	271
	**Dietary information factors**		
1	Drinks	Drinking sugar-containing drinks (Coke, Fanta, soda, fruit juice, other sweet/sugar-containing drinks) three or more times a week	271
2	Eating fast foods	Eating fast foods such as pizzas, hamburgers, deep-fried foods (e.g., singara, samosa, Mughlai paratha, etc.) outside three or more times a week	271

Note: Clinical factors = anthropometric + biochemical measurements.

**Table 2:** PHC data range validation chart.

Parameter (Independent Variables)		Healthy	Caution	Affected	Emergent
Height (cm)					
Weight (kg)					
BMI		<25	≥25, <30	≥30, <35	≥35
Waist (cm)	Male	<90.0	≥90.0	NA	NA
	Female	<80.0	≥80.0	NA	NA
Hip (cm)					
Waist/hip ratio	Male	<0.90	≥0.90	NA	NA
	Female	<0.85	≥0.85	NA	NA
Body temperature (°F)		<98.6	≥98.6, <99.5	≥99.5	NA
Oxygenation of blood (%)		≥96	≥93, <96	≥90, <93	<90
Blood pressure (mmHg)	Systolic	<130	≥130, <140	≥140, <180	≥180
	Diastolic	<85	≥85, <90	≥90, <110	≥110
Blood sugar (mmol/dL)	RBS	<7.78	≥7.78, <11.11	≥11.11, <16.67	≥16.67
	FBS	<5.56	≥5.56, <7.0	≥7.0, <11.11	≥11.11
Blood hemoglobin (g/dL)		≥12.0	≥10.0, <12.0	≥8.0, <10.0	<8.0
Pulse rate (bpm)		≥60, <100	≥50, <60 OR ≥100, <120	<50 OR ≥120	NA
Arrhythmia		Normal		Others	
Blood cholesterol (mg/dL)		≤200.0	>200.0, ≤225.0	>225.0, <240.0	≥240.0
Blood uric acid (mg/dL)	Male	>3.5, ≤7.0		>7.0, <8.0	≥8.0
	Female	>2.4, ≤6.0		>6.0, <7.0	≥7.0

Note: NA means not applicable.

**Table 3:** Summary statistics of the selected continuous predictors (*n* = 271).

Number	Variables	Minimum	Maximum	Mean ± Std. Deviation
1	Age	34	77	49.61 ± 7.39
2	Height (cm)	140.00	184.00	163.05 ± 7.45
3	Weight (kg)	44.20	114.40	67.52 ± 10.06
4	BMI (kg/m^2^)	18.39	40.53	25.37 ± 3.20
5	Waist (cm)	63.60	118.00	90.24 ± 7.80
6	Hip (cm)	80.00	127.00	94.54 ± 6.29
7	Waist/hip ratio	0.64	1.11	0.96 ± 0.06
8	Body temperature (°F)	92.12	99.64	96.07 ± 1.15
9	SpO2	93	99	97.67 ± 1.17
10	Systolic BP (mmHg)	92	180	126.68 ± 14.88
11	Diastolic BP (mmHg)	59	108	81.71 ± 8.43
12	Pulse rate (bpm)	51	123	80.27 ± 11.66
13	Blood uric acid (mg/dL)	3.10	11.00	6.63 ± 1.54

Note: SpO2 means oxygenation of blood.

**Table 4:** Summary statistics of the selected categorical predictors.

No.	Categorical Variables	Description	Categories/Levels	Frequency	%
1	Gender	Gender of the participant	Male = 1;	225	83.0
			female = 0	46	17.0
2	Education	Education completed by the participant	1 = No education (no school entered);	10	3.7
			2 = Primary school completed;	30	11.1
			3 = Secondary school completed;	11	4.1
			4 = High school completed;	23	8.5
			5 = Vocation school completed;	1	0.4
			6 = College/university completed;	63	23.2
			7 = Higher (master or doctor) completed	133	49.1
3	Drinks	Drinking sugar-containing drinks (Coke, Fanta, soda, fruit juice, other sweet/sugar-containing drinks) three or more times a week	2 = Yes;	26	9.6
			1 = No	245	90.4
4	Eating fast foods	Eating fast foods such as pizzas, hamburgers, deep-fried foods (e.g., singara, samosa, Mughlai paratha, etc.) three or more times a week	2 = Yes;	49	18.1
			1 = No	222	81.9
5	Health status	Overall health condition	1 = healthy;	2	0.7
			2 = caution;	80	29
			3 = affected;	122	45
			4=emergent	67	25

Note: Singara, samosa, Mughlai paratha, etc., are popular deep-fried fast foods in Bangladesh.

**Table 5:** Results of the multinomial logistic regression estimation (*n* = 269).

Health Status	Factors Name	Estimate B	Std. Error	Z Value	Pr(>|z|)	Exp(B)
Affected	Intercept	20.09	0.01	3243.46	<2.2 × 10^−16^ ***	5.26 × 10^8^
	Age	0.05	0.03	1.76	0.08	1.04
	Gender	−1.60	0.77	−2.09	0.04 *	0.21
	Education	−0.04	0.09	−0.38	0.71	0.97
	Height	−0.17	0.11	−1.53	0.13	0.85
	Weight	0.19	0.14	1.39	0.17	1.21
	Waist	0.17	0.05	3.47	0.00 ***	1.19
	BMI	−0.43	0.37	−1.15	0.26	0.66
	Hip	−0.21	0.07	−3.01	0.01 **	0.82
	WHR	−12.42	0.01	−1698.74	<2.2 × 10^−16^ ***	4.04 × 10^−6^
	SpO2	0.23	0.14	1.66	0.09	1.25
	Systolic BP	0.03	0.02	1.54	0.13	1.04
	Diastolic BP	0.07	0.4	2.08	0.04 *	1.08
	Drinks	−0.63	0.58	−1.09	0.28	0.54
	Eating fast food	0.27	0.49	0.54	0.60	1.31
	Body temperature	−0.15	0.16	−0.94	0.35	0.87
	Pulse rate	0.02	0.02	1.15	0.26	1.02
	Blood uric acid	0.60	0.15	3.96	7.35 × 10^−5^ ***	1.82
Emergent	Intercept	98.04	0.01	10298.82	<2.2 × 10^−16^ ***	3.77 × 10^42^
	Age	0.03	0.04	0.79	0.44	1.03
	Gender	−3.55	1.04	−3.44	0.00 ***	0.03
	Education	−0.06	0.14	−0.41	0.68	0.95
	Height	−0.91	0.16	−5.70	1.21 × 10^−8^ ***	0.41
	Weight	1.01	0.19	5.20	1.92 × 10^−7^ ***	2.73
	Waist	−0.32	0.08	−4.38	1.18 × 10^−5^ **	0.74
	BMI	−2.77	0.54	−5.17	2.30 × 10^−7^ ***	0.07
	Hip	0.42	0.10	4.34	1.42 × 10^−5^ ***	1.51
	WHR	42.59	0.02	4005.93	<2.2 × 10^−16^ ***	3.12 × 10^−18^
	SpO2	0.07	0.19	0.34	0.74	1.08
	Systolic BP	0.07	0.03	2.48	0.01 *	1.07
	Diastolic BP	−0.01	0.05	−0.15	0.89	0.99
	Drinks	−0.52	0.96	−0.54	0.59	0.60
	Eating fast food	0.29	0.77	0.36	0.72	1.33
	Body temperature	−0.32	0.23	−1.43	0.16	0.73
	Pulse rate	0.03	0.03	0.97	0.34	1.03
	Blood uric acid	2.40	0.30	7.99	1.27 × 10^−15^ ***	11.02

Note: ***, ** and * indicate the variables significantly associated with health status at *p* values 0.001, 0.01, 0.05 and 0.1, respectively. The reference category is “caution”. Residual deviance = 373.38.
